# Memory enhancing effects of BPN14770, an allosteric inhibitor of phosphodiesterase-4D, in wild-type and humanized mice

**DOI:** 10.1038/s41386-018-0178-6

**Published:** 2018-08-14

**Authors:** Chong Zhang, Ying Xu, Anirudh Chowdhary, David Fox, Mark E. Gurney, Han-Ting Zhang, Benjamin D. Auerbach, Richard J. Salvi, Mingxin Yang, Gaowen Li, James M. O’Donnell

**Affiliations:** 1 Department of Pharmacology and Toxicology, Jacobs School of Medicine and Biomedical Sciences, University at Buffalo, State University of New York, Buffalo, NY 14214 USA; 2 Department of Pharmaceutical Sciences, School of Pharmacy and Pharmaceutical Sciences, University at Buffalo, State University of New York, Buffalo, NY 14214 USA; 3 Tetra Discovery Partners Inc., Grand Rapids, MI 49503 USA; 4 Beryllium Discovery Corp., Bainbridge Island, WA 98110 USA; 5 Departments of Behavioral Medicine and Psychiatry & Physiology, Pharmacology, and Neuroscience, West Virginia University Health Sciences Center, Morgantown, WV 26506 USA; 6 Center for Hearing & Deafness, University at Buffalo, State University of New York, Buffalo, NY 14214 USA

## Abstract

Inhibitors of phosphodiesterase-4 (PDE4) have beneficial effects on memory in preclinical and clinical studies. Development of these drugs has stalled due to dose-limiting side effects of nausea and emesis. While use of subtype-selective inhibitors (i.e., for PDE4A, B, or D) could overcome this issue, conservation of the catalytic region, to which classical inhibitors bind, limits this approach. The present study examined the effects of BPN14770, an allosteric inhibitor of PDE4D, which binds to a primate-specific, N-terminal region. In mice engineered to express PDE4D with this primate-specific sequence, BPN14770 was 100-fold more potent for improving memory than in wild-type mice; meanwhile, it exhibited low potency in a mouse surrogate model for emesis. BPN14770 also antagonized the amnesic effects of scopolamine, increased cAMP signaling in brain, and increased BDNF and markers of neuronal plasticity associated with memory. These data establish a relationship between PDE4D target engagement and effects on memory for BPN14770 and suggest clinical potential for PDE4D-selective inhibitors.

## Introduction

Genetic studies in model organisms and recent use of exon sequencing in rare human disorders identify the cyclic AMP (cAMP)–protein kinase A (PKA)–cAMP-response element binding protein (CREB) pathway as fundamental to early and late stages of memory formation [1]. Studies of learning mutations in *Drosophila* identified the *dunce* and *rutabaga* mutations which later were shown to be mutations in phosphodiesterase-4 (PDE4), in the case of *dunce* [2, 3], and calcium/calmodulin-dependent adenylyl cyclase, in the case of *rutabaga* [4]. In mammals, calcium/calmodulin-dependent adenylyl cyclase acts downstream of the *N*-methyl-d-aspartate (NMDA) receptor to trigger cAMP synthesis in response to calcium influx [5]. This activates the PKA–CREB pathway which is responsible for the earliest, transient stages of memory such as long-term potentiation (LTP) in the hippocampus [6] and also changes in protein synthesis and gene expression that lead to memory consolidation [7], increased expression of brain-derived neurotrophic factor [8], and neurogenesis [9, 10].

The *Drosophila dunce* mutation is a null mutation of PDE4 that disrupts memory by allowing the unregulated accumulation of cAMP within neurons, thereby destroying the spatial and temporal patterning of cAMP signaling [11, 12]. The *Drosophila* genome contains only a single PDE4 gene, while in vertebrates this has been expanded into a gene family containing four subtypes, PDE4A, B, C, and D [13]. In humans, ultra-rare missense mutations in PDE4D cause acrodysostosis type 2, with or without hormone resistance (ACRDYS2), a neurodevelopmental disorder associated with intellectual disability, speech and psychomotor retardation, brachydactyly, facial dysostosis, and spinal stenosis [1, 14–17]. The PDE4 enzymes are distinguished from other PDE families by the presence of a pair of upstream regulatory domains known as “upstream conserved regions” (i.e., UCR1 and UCR2) [18]. PDE4 enzyme activity is regulated by the opening and closing of UCR2 across the catalytic site [19], while UCR1 is required for assembly of dimeric forms of the PDE4 enzymes [20, 21]. The activity of the dimeric form is upregulated by PKA phosphorylation of UCR1 [22–24], while activation of the PKA–CREB pathway upregulates PDE4 gene expression [25]. ACRDYS2 mutations affect the UCR1 PKA phosphorylation site [17], thereby preventing upregulation of enzyme activity in response to cAMP signaling, or affect contact residues between UCR2 and the catalytic domain, thereby preventing enzyme inactivation through closure of UCR2 [26]. Thus, PDE4D enzymatic activity is dynamically regulated by signaling through the PKA–CREB pathway in a manner critical to normal cognitive function.

The absolute amino acid sequence conservation of the PDE4 catalytic site, to which classic enzyme inhibitors bind, across the four PDE4 subtypes has made it difficult to develop subtype-selective inhibitors [27]. However, a single amino acid difference in UCR2, a phenylalanine in PDE4D and a tyrosine in PDE4A, B, and C, has allowed the design of PDE4D subtype-selective allosteric inhibitors [19]. PDE4D allosteric inhibitors bind in the catalytic site and complete a hydrophobic surface that allows closure of the amphipathic UCR2 regulatory helix in which hydrophobic residues are oriented towards the catalytic site. Closure of UCR2 inhibits the access of cAMP to the catalytic site and consequently enzymatic activity. The binding pose of rolipram, a widely studied allosteric inhibitor of PDE4, accommodates either phenylalanine or tyrosine when UCR2 is closed over the active site. By contrast, subtype-selective PDE4D allosteric inhibitors are designed to accommodate the phenylalanine while clashing with UCR2 containing a tyrosine (i.e., for the PDE4A, B, and C subtypes) as the tyrosine protrudes more deeply into the active site. The phenylalanine that distinguishes PDE4D UCR2 from PDE4A, B, and C is unique to primates. As the species difference lowers the *K*_M_ for cAMP, we speculate a PDE4D enzyme with greater processivity at low substrate concentration may have been selected during evolution as part of the gene set for intelligence in the primate brain.

Based on the unique binding pose of BPN14770 to primate PDE4D, we humanized the mouse PDE4D gene by knocking into C57bl/6 mouse embryonic stem cells a single-nucleotide substitution that replaces UCR2 tyrosine 271 by phenylalanine. By comparing their response to BPN14770 against wild-type, littermate controls, the humanized PDE4D mice therefore provide a unique and powerful genetic tool with which to asses PDE4D target engagement by BPN14770, and thereby, the ability of a PDE4D allosteric inhibitor to modulate neurochemical and cognitive biomarkers of PKA–CREB pathway outflow.

## Materials and methods

Methods and materials are described in detail in the Supplementary Materials and methods.

### Compounds

BPN14770 was synthesized as previously described [28]. Rolipram was purchased from Enzo Life Sciences (Farmingdale, NY). H-89 and scopolamine were purchased from Sigma-Aldrich (St. Louis, MO). [^3^H]-rolipram was purchased from PerkinElmer (Waltham, MA).

### Enzymes

PDE4D residue numbering is based on the reference PDE4D7 isoform, NCBI (National Center for Biotechnology Information) Reference Sequence: NP_001159371.1. Methods used to generate synthetic genes for human and mouse PDE4 subtypes and isoforms were as described in Burgin et al. [19]. Synthetic genes were engineered with carboxyl- or amino-terminal hexahistidine tags for Baculovirus-infected insect cell expression and purification. Vendors were Beryllium Discovery Corp (Bainbridge Island, WA) and Proteos Inc. (Kalamazoo, MI).

### Animals

C57bl/6 mice homozygous for the humanized PDE4D gene were generated by inGenious Targeting Laboratory (Ronkonkoma, NY) and maintained at the University at Buffalo. All behavioral tests were performed during 9:30 am–16:30 pm and in accordance with the “NIH Guide for the Care and Use of Laboratory Animals” (revised 2011) and were approved by the Institutional Animal Care and Use Committee of the University at Buffalo.

### PDE4 biochemical assays

Kinetic assay of cAMP hydrolysis by purified PDE4 enzymes is measured by coupling the formation of the PDE4 reaction product 5′-adenosine monophosphate (AMP) to the oxidation of reduced nicotinamide adenine dinucleotide (NADH). This is accomplished with three coupling enzymes (yeast myokinase, pyruvate kinase, and lactate dehydrogenase), and allows fluorescent determination of reaction rates [19]. To measure inhibition of PDE4 enzymes by test compounds, data were percent normalized relative to controls and are presented as percent inhibition.

### [^3^H]-rolipram radioligand binding

Mice were killed by decapitation, and the brains were immediately dissected on ice and homogenized in binding buffer using a Polytron homogenizer (Brinkman Instruments, Westbury, NY). The membrane fraction of the homogenate was obtained by centrifugation at 15,000 × *g* for 15 min, and resuspension of the pellet in the binding buffer. The method was modified by Zhao and coworkers [29, 30] based on Schneider’s earlier publication. Mouse brain membrane preparations containing 200 to 300 μg of protein were used for co-incubation with various concentrations of BPN14770 and/or [^3^H]-rolipram.

### Behavioral tests

#### Y-maze spontaneous alternation test

The test consists of a single 5 min trial, in which the mouse was allowed to explore all three arms of the Y-maze [31]. Spontaneous alternation (%) was defined as consecutive entries in three different arms, divided by the number of possible alternations (total arm entries minus 2).

#### Novel object recognition

The novel object recognition test was performed as described elsewhere [32, 33]. Briefly, the task procedure consists of three phases: habituation phase on day 1 for 10 min, training (T1) phase on day 2 for 5 min, and testing (T2) phase on day 3 for 5 min. The duration each animal spent exploring the objects was recorded. Time spent exploring the identical objects in T1 was recorded as *a*_1_ and *a*_2_; time spent exploring the familiar and the novel objects in T2 was recorded as “*a*” and “*b*”, respectively. The following variables were calculated: *e*1 *=* *a*_1_ *+* *a*_2_*, e2* *=* *a* *+* *b*, the relative discrimination index *d*2 *=* *(b*–*a)/e*2.

#### Ketamine/xylazine test

Mice were dosed with BPN14770 or vehicle (per os (PO)), or rolipram (1 mg/kg, intraperitoneal (IP)). After 30 min or 15 min (for PO and IP doses, respectively), mice were injected (IP) with a mixture of ketamine (80 mg/kg) and xylazine (10 mg/kg). The duration of anesthesia was determined as the time between the loss and return of the righting reflex, and was used as an endpoint to measure the duration of anesthesia.

### cAMP assay

Mice were treated with various doses of BPN14770 or vehicle and killed 1 h after dosing by rapid decapitation, and brains were immediately dissected on ice, flash frozen in liquid nitrogen, and stored at −80 °C. On the day of assay, frozen brain samples were analyzed using the cAMP complete ELISA kit (Enzo Life Sciences, Farmingdale, NY) according to the assay protocol [34].

### Hippocampal CA1 field recording

Transverse hippocampal slices at 350 μm thickness were prepared by a compresstome slicer (Precisionary Instruments, Greenville, NC, USA) in the high-sucrose dissection buffer. LTP was induced by high-frequency stimulation (HFS) consisting of a single train of 100 pulses delivered at 100 Hz. This HFS paradigm was used to induce a short-lasting form of LTP in the control slices, so that any additional potentiation of LTP drug treatment could be observed. BPN14770 was dissolved in dimethyl sulfoxide (DMSO) and diluted into artificial cerebrospinal fluid (aCSF) with a final DMSO concentration of 0.01%, and was bath-applied in the flowing aCSF from −10 min to +30 min relative to the time of HFS [35].

### Immunoblot analyses

Hippocampal tissues were treated with lysis buffer for protein extraction. Then, 10–30 μg protein was separated using gel electrophoresis and transferred to polyvinylidene difluoride membranes, blocked with blocking buffer, and incubated with primary antibodies overnight at 4 °C. Labeled protein bands were detected using the enhanced chemiluminescence method and quantified using Quantity One 1-D Analysis Software.

### Statistical analyses

All data are presented as means ± SEM and were analyzed using GraphPad Prism. For multiple comparisons, data were analyzed using one-way or two-way analysis of variance (ANOVA) with Dunnett’s correction for multiple comparisons. Student’s *t*-test was used for pairwise comparisons between vehicle-treated and positive control drug-treated or scopolamine-treated groups. Statistical significance was set at *p* < 0.05.

## Results

### Design of BPN14770 and humanized mice

**Fig. 1:**
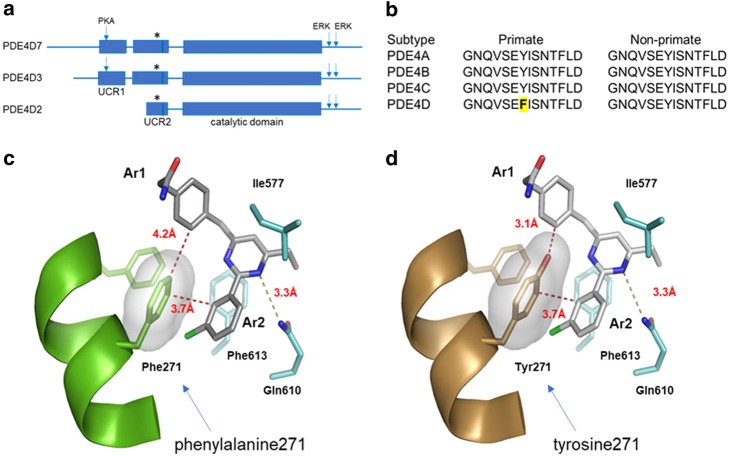
Binding pose of a PDE4D-NAM in the allosteric site. **a** Relative size and position of UCR1, UCR2, and the catalytic domain in the PDE4D polypeptide. PDE4D7 and PDE4D3 contain the UCR1 and UCR2 helices needed for dimerization through formation of a four-helix bundle. PDE4D2 lacks these helices, and so is active as a monomer. Arrows indicate the locations of the PKA phosphorylation site on UCR1 and the ERK phosphorylation sites distal to the catalytic domain. **b** The amino acid sequence of the UCR2 helix that closes over the active site. The sequence is conserved between PDE4D subtypes and across species with the exception of the key phenylalanine (asterisk in (**a**), bold and yellow highlighted in (**b**)) that is present in primate PDE4D UCR2. **c** A co-crystal structure of BPN5004 bound in the PDE4D allosteric site (PDB ID: 6BOJ). UCR2 is shown as a green ribbon structure, while catalytic domain residues are colored cyan. The pyrimidine core is clamped above and below the plane of the ring between an active site isoleucine (Ile577) and a phenylalanine (Phe613) while forming a 3.3 Å hydrogen bond to the invariant PDE active site glutamine (Gln610). Modeling of BPN14770 in the 6BOJ structure suggests that it has a similar binding pose to BPN5004. Closure of UCR2 allows the key phenylalanine selectivity residue (Phe271) to interact with the two aromatic arms of the allosteric inhibitor through an edge-on, π–π interaction to Ar1 (4.2 Å) and π–π stacking with Ar2 (3.7 Å). **d** Modeling of the tyrosine replacement suggests the deeper projection of the tyrosine hydroxyl group will clash sterically with the edge-on Ar2 phenyl ring (3.1 Å) and the binding energy contributed by the edge-on, π–π interaction will be lost. The larger bulk of the tyrosine hydroxyl relative to the phenylalanine is visualized by the light gray surface rendition with transparency

PDE4D7, a representative dimeric form of PDE4D, is highly conserved across species with >99% amino acid sequence conservation across the 748 amino acid length of the polypeptide. The core amino acid sequence of PDE4D7 from UCR1 through the end of the catalytic domain is absolutely conserved across all species except for the phenylalanine in UCR2 unique to primate (phenylalanine 271) which in non-primate species is a tyrosine (Fig. 1a, b). Assembly of dimeric forms of PDE4 is through a four-helix bundle formed from the last helix of UCR1 and the first helix of UCR2 [20, 21]. The third helix of UCR2 opens and closes over the active site and contains the key phenylalanine selectivity residue [19].

X-ray crystallography shows that the binding pose of BPN14770 to PDE4D in the allosteric site highly resembles that of BPN5004 to PDE4D (Supplementary table S1, Fig. 1c, d). PDE4 allosteric inhibitors share a common pharmacophore that allows the compound to engage UCR2 [19]. A central aromatic core is clamped between a pair of hydrophobic active site residues and donates an electron pair to a hydrogen bond formed with an invariant, active site glutamine [27]. The central core is decorated with a pair of projecting, aromatic arms denoted as Ar1 and Ar2 [19, 36]. The key phenylalanine selectivity residue projects from UCR2 into the active site where it makes an edge-to-edge or edge-on π–π interaction with the Ar1 aromatic ring and a face-to-face π stacking interaction with the aromatic Ar2 ring. In PDE4B, the tyrosine hydroxyl projects deeper into the active site, thereby creating a steric clash with the Ar1 aromatic ring of the allosteric inhibitor, and the additional binding energy contributed by the π–π interaction is lost.

BPN14770 is equipotent against two different dimeric forms of PDE4D (PDE4D7 half-maximal inhibitory concentration (IC_50_) = 7.8 ± 1.8 nM; PDE4D3 IC_50_ = 7.4 ± 2 nM), but is 16-fold less potent against a monomeric form of PDE4D lacking the UCR1 dimerization domain (PDE4D2 IC_50_ = 127 ± 1.2 nM; Table 1). The compound is 120-fold more potent against an activated form of the PDE4D dimer containing a mutation in UCR1 to mimic PKA phosphorylation as compared to the basal form of the PDE4D dimer. BPN14770 may have greater potency against the dimeric forms of PDE4D due to pre-organization of UCR2 in *trans* across the active site [21]. Translation of PDE4D2 begins at a methionine internal to UCR2, and hence the protein lacks the helices in UCR1 and UCR2 needed for dimerization (Fig. 1a). The truncated UCR2 of PDE4D2 is linked to the catalytic domain by a disordered spacer of 40 amino acids, and hence it is not clear how that geometry may accommodate pre-organization of UCR2 across the active site.

**Table 1:** Inhibitory potency of BPN14770 against human and mouse PDE4 subtypes and isoforms

Species	Enzyme	Form	Mutations^a^	BPN14770 IC_50_ (nM)	Rolipram IC_50_ (nM)
Human	PDE4D7	Dimer	Native	1018 ± 239	675 ± 8.5
Human	PDE4D7	Dimer	S129D(PKA), S654A(ERK), S656A(ERK)	7.8 ± 1.8	32 ± 10
Human	PDE4D3	Dimer	S54D(PKA), S579A(ERK), S581A(ERK)	7.4 ± 2	29 ± 11
Human	PDE4D2	Monomer	S413A(ERK), S415A(ERK)	127 ± 1.2	142 ± 51
Human	PDE4B1	Dimer	S133D(PKA), S659A(ERK), S661A(ERK)	2013 ± 256	175 ± 36
Mouse	PDE4D7	Dimer	S129D(PKA)	133 ± 18	30 ± 17
Mouse	PDE4D7	Dimer	S129D(PKA), Y271F(UCR2)	2.9 ± 0.3	36 ± 2.5
Mouse	PDE4B3	Dimer	S133D(PKA)	2124 ± 527	223 ± 34

^a^PKA-activating mutation of serine to aspartic acid to mimic phosphorylation; ERK-null mutation of serine to alanine to prevent phosphorylation; UCR2 replacement of tyrosine by phenylalanine to humanize the mouse PDE4D UCR2

BPN14770 is 17-fold less potent against mouse PDE4D7 (IC_50_ = 133 ± 18 nM), a dimeric form, and only 16-fold selective for mouse PDE4B (PDE4B3 IC_50_ = 2,124 ± 527 nM). Mutation of the key selectivity residue, mouse UCR2 tyrosine 271 to phenylalanine, increases BPN14770 potency to an IC_50_ = 2.9 ± 0.3 nM, a 46-fold increase in potency with 730-fold selectivity against mouse PDE4B3 (Table 1).

For comparison, the binding pose of rolipram, an atypical PDE4 inhibitor that also engages UCR2, accommodates either the UCR2 phenylalanine or the UCR2 tyrosine [19]. Nonetheless, rolipram has modest 6–7-fold selectivity for mouse or human PDE4D over PDE4B (Table 1).

The difference in potency of selective PDE4D allosteric inhibitors against the wild-type versus humanized mouse PDE4D enzyme prompted us to engineer a humanized PDE4D mouse on a C57bl/6 inbred strain background. This allowed us to assess BPN14770 engagement of the PDE4D target by comparing the potency of the compound in mice homozygous for the engineered, humanized PDE4D gene versus littermate control mice carrying the wild-type, native mouse PDE4D gene using neurochemical and cognitive biomarkers of PKA–CREB pathway activation. Significantly greater potency, e.g., 100-fold, of BPN14770 in humanized vs. wild-type mice for a particular effect would suggest predominant mediation by the PDE4D subtype.

### Inhibition of [^3^H]-rolipram binding by BPN14770 to membranes prepared from wild-type and humanized PDE4D mouse brains

**Fig. 2:**
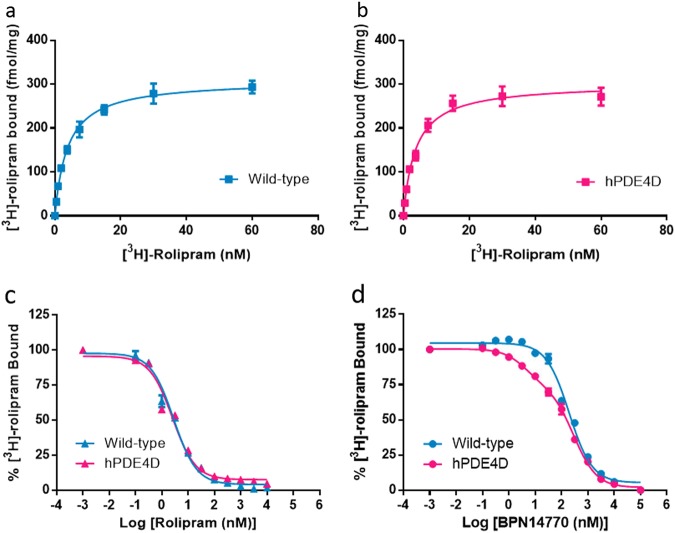
BPN14770 differentiated the high affinity binding site in humanized PDE4D (hPDE4D) mouse brain preparations. **a**, **b** Saturation binding performed using increasing concentrations of [^3^H]-rolipram with wild-type (**a**) and humanized PDE4D (**b**) mouse brain membrane preparations. **c** Displacement of [^3^H]-rolipram binding by rolipram in the wild-type and humanized PDE4D mouse brain preparations, which were both described by a one-site binding model. **d** Displacement of [^3^H]-rolipram binding by BPN14770 in the wild-type and humanized PDE4D mouse brain preparations, of which humanized PDE4D was better described by a two-site binding model. Statistical details are summarized in Table 2

PDE4, including all subtypes, not only control the overall level but also the subcellular compartmentalization of cAMP, due at least in part to their intracellular localization [37]. The association of changed behavior to altered binding of PDE4 with the inhibitors in the brain membrane fraction was shown in a previous study [38]. Saturation binding of [^3^H]-rolipram has been used to quantify PDE4 binding sites in mouse brain membrane fractions [30]. Since rolipram has little selectivity for PDE4 subtypes, total binding should represent the sum of binding to PDE4A, B, and D, the primary PDE4 subtypes expressed in brain [39, 40]. [^3^H]-rolipram bound with high affinity to membranes prepared from brains of wild-type (*K*_d_ = 3.84 nM) and humanized PDE4D (*K*_d_ = 3.72 nM) mice (Fig. 2a, b). There was no change in total binding to wild-type (*B*_max_ = 309.2 fmol/mg protein) as compared to humanized PDE4D (*B*_max_ = 301.8 fmol/mg protein) brain samples. Unlabeled rolipram inhibited [^3^H]-rolipram binding with high affinity and the data were adequately fit by a one-site model with *K*_i_ = 1.99 ± 0.11 nM for wild-type mice and 1.66 ± 0.07 nM for humanized PDE4D mice (Fig. 2c, Table 2). Thus, expression of humanized PDE4D in mouse brain did not alter high affinity [^3^H]-rolipram binding nor did genetic manipulation of the humanized mouse alter PDE4 gene expression as *B*_max_ was unchanged.

**Table 2:** Inhibition of [^3^H]-rolipram binding to mouse brain membranes by BPN14770 or rolipram

		One-site	Two-site		Hill slope	*F*	Preferred model
		*K*_i_ (nM)	*K*_i1_ (nM)	*K*_i2_ (nM)			
Rolipram	Wild-type	1.99 ± 0.11	0.75 ± 0.33	34.1 ± 26.83	−0.75 ± 0.08	2.92	One-site
	hPDE4D	1.66 ± 0.07	0.39 ± 0.07	6.10 ± 0.79	−0.71 ± 0.02	1.91	One-site
BPN14770	Wild-type	124.4 ± 14.1	79.7 ± 17.09	622.4 ± 109.8	−0.89 ± 0.06	0.98	One-site
	hPDE4D	82.1 ± 6.03	2.80 ± 1.08	186.3 ± 19.0	−0.56 ± 0.02	11.25	Two-site

*K*_i_ values, Hill slope, and *F*-ratio were calculated by non-linear regression. Binding to a single site was assumed unless the data were better described by a two-site model, which is characterized by a small Hill slope and large residual sum of square (*F*-ratio). Statistical difference was set at *p* < 0.001. Data shown are from three independent experiments (mean ± SEM)

To assess BPN14770 engagement of the PDE4D target, inhibition of [^3^H]-rolipram binding by BPN14770 was compared in brain membrane preparations from wild-type and humanized PDE4D mice (Fig. 2d). This revealed the presence of a high affinity binding site for BPN14770 in the humanized PDE4D mouse brain that was not present in wild-type mice. Inhibition of [^3^H]-rolipram binding to humanized PDE4D mouse brain membranes was better fit by a two-site model (*K*_i1_ = 2.80 ± 1.08 nM; *K*_i2_ = 186.3 ± 19.0 nM, *F* = 11.25, *p* < 0.001) whereas in wild-type mice, inhibition by BPN14770 was adequately fit by a one-site model (*K*_i_ = 124.4 ± 14.1 nM, *F* = 0.98), Table 2. In the humanized PDE4D mice, the high affinity, 2.80 nM *K*_i1_ sites accounted for 26.7% of total binding. Thus, expression of humanized PDE4D in mouse brain is associated with the de novo appearance of high affinity binding sites for BPN14770. Although in wild-type mice, BPN14770 should show modest 16-fold selectivity for PDE4D over PDE4B, a two-site model was not preferred over a one-site model (Table 2).

### Brain cAMP concentrations in mice treated with BPN14770

**Fig. 3:**
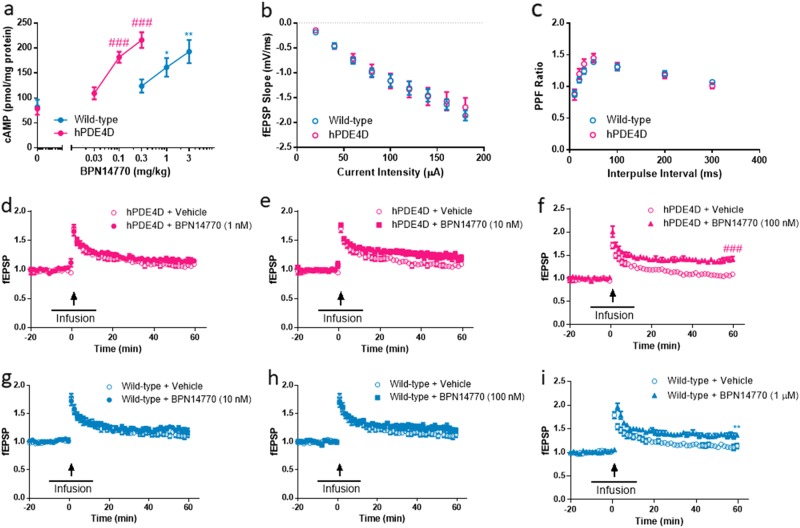
BPN14770 elevates brain cAMP and facilitates hippocampal long-term potentiation (LTP) in wild-type and humanized PDE4D mice. **a** cAMP concentrations in both wild-type and humanized PDE4D mouse brains were significantly increased after treatment with BPN14770. Data are presented as mean ± SEM (*n* = pooled analysis of 3–5 males and 3–5 females per group); *p *<* 0.05 and **p < 0.01 (Dunnett’s multiple comparison) versus vehicle-treated wild-type mice; ^###^*p* < 0.001 (Dunnett’s multiple comparison) versus vehicle-treated hPDE4D mice. **b** Summary of input–output curves generated by applying increasing intensity of stimuli and measuring the initial slopes of fEPSP for both wild-type and humanized PDE4D mouse slices. **c** Paired-pulse facilitation (PPF) was measured by applying two consecutive stimuli with varying interpulse intervals and obtaining the ratio of the second fEPSP slope to the first, for both wild-type and humanized PDE4D mice slices. **d**–**f** Induction of LTP in humanized PDE4D mice hippocampal slices mediated by increasing concentrations of BPN14770 (1 nM, 10 nM, and 100 nM). The time course of infusion is indicated by the horizontal bar and the time of tetanic stimulation is indicated by the arrow. **g**–**i** Induction of LTP in wild-type hippocampal slices mediated by increasing concentrations of BPN14770 (10 nM, 10 nM, and 1 µM). Data are presented as mean ± SEM (*n* = 8); ^###^*p* < 0.001 (Student’s *t*-test) versus vehicle-treated control hPDE4D slices; **p < 0.01 (Student’s *t*-test) versus vehicle-treated control wild-type slices

Rolipram previously has been shown to increase mouse brain cAMP after acute dosing [41, 42]. Therefore, we measured brain cAMP in male and female mice 1 h after oral administration of BPN14770 (Fig. 3a), a time-point at which adequate drug distribution to the brain is achieved. BPN14770 oral bioavailability is 70–80% in mice with a *t*_1/2_ in plasma and brain of approximately 8–10 h. The brain to plasma ratio is 0.4–0.42 in C57bl/6 mice based on *C*_max_ or area under the curve (data not shown). Baseline brain cAMP is similar in the wild-type and humanized PDE4D mice. BPN14770 increased brain cAMP in both types of mice with humanized PDE4D mice being more sensitive to the effects of the compound (one-way ANOVA; wild-type: *F*(3, 12) = 7.169, *p* = 0.0051; hPDE4D: *F*(3, 18) = 23.20, *p* < 0.0001). The minimum effective dose (MED) of BPN14770 providing a statistically significant increase in brain cAMP is 0.1 mg/kg PO in humanized mice (*p* < 0.001) and 1 mg/kg PO in wild-type mice (*p* < 0.05). Brain cAMP is increased almost three-fold in the humanized PDE4D mice at 0.3 mg/kg PO (*p* < 0.001), a dose that is ineffective in wild-type mice. The ten-fold potency difference between the mouse genotypes suggests that the effects of BPN14770 are due to inhibition of PDE4D (i.e., beginning at lower doses) as well as PDE4A and B. Little contribution of PDE4C inhibition in elevating cAMP concentrations is expected due to its low expression levels in the brain [37].

### Effects of BPN14770 on LTP in hippocampal slices

The PKA–CREB pathway plays a critical role in regulating LTP in the hippocampus, one of the initial, transient changes in synaptic plasticity that underlies early stages of memory [43]. Multiple studies have shown that rolipram augments LTP [44], which consists of two temporally distinct phases, an early phase (E-LTP) that lasts 1–3 h and does not require gene transcription and protein synthesis, and a late phase (L-LTP) that can be recorded 6–8 h in vitro and is transcription and translation dependent. Duration of LTP depends on complex factors such as the form and intensity of stimulation and the absence or presence of a pharmacological manipulation [45]. Wild-type and humanized PDE4D mouse hippocampal slices did not differ in basal synaptic transmission (postsynaptic responses to increasing stimulation intensities) or paired-pulse facilitation (PPF), an index of short-term plasticity (Fig. 3b, c). While hippocampal slices exhibited short-lasting enhancement of field excitatory postsynaptic potential (fEPSP), increasing doses of BPN14770 potentiated such effects, to a point where LTP did not decay during the time of recording, in both wild-type and humanized PDE4D slices (Fig. 3d–f, g–i). Augmentation of E-LTP occurred at a minimum bath concentration of 100 nM BPN14770 with slices from humanized PDE4D mice, and at 1 µM BPN14770 with slices from wild-type mice, respectively (Fig. 3f, i). These results suggest that PDE4D contributes to this effect of BPN14770; however, given the smaller potency difference (as compared to the difference between the *K*_i_ for the high affinity binding site in humanized PDE4D brains and the *K*_i_ in wild-type mice), other PDE4 subtypes likely also contribute. An important role of PDE4D, rather than PDE4B, in mediating E-LTP was demonstrated earlier by knockout mouse models [46]. However, this is the first time that a PDE4D allosteric modulator was used to reveal the role of PDE4D in regulating early stages of hippocampal neuroplasticity.

### Effects of BPN14770 on memory

Both early and late memory processes are dependent upon activation of the PKA–CREB pathway with rolipram and multiple other PDE4 inhibitors showing cognitive benefit in diverse memory models [47]. Y-maze and novel object recognition (NOR) were used to assess short- and long-term memory formation dependent upon the PKA–CREB pathway in wild-type and humanized PDE4D mice and the dose response to BPN14770. In the Y-maze test, alternation is defined as the number of arm choices that differed from the previous two choices. For efficient alternation, mice need to use spatial and working memory, and continue to explore the least recently visited arm. In the NOR, mice are exposed to a pair of identical objects for 5 min, and then later to the same object and a novel object. If the object seen in the first trial is recognized as familiar, the mice are expected to spend more time exploring the novel object compared to the familiar object, given their innate preference for novelty. In the NOR, by varying the inter-trial interval, the same paradigm can be used to explore short- or long-term memory.

**Fig. 4:**
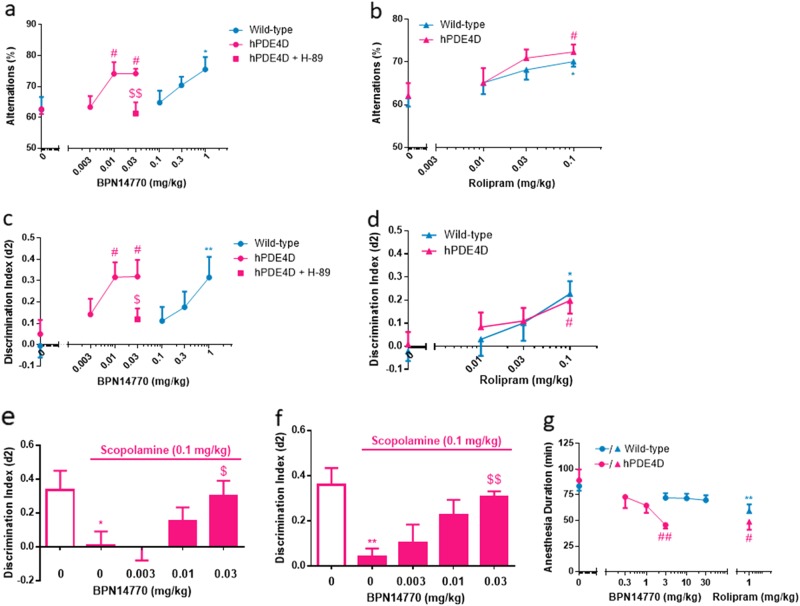
BPN14770 improves cognitive performance in wild-type and humanized PDE4D mice in Y-maze and NOR without causing significant reduction in ketamine/xylazine-induced anesthesia at cognitive-beneficial doses. **a** BPN14770 treatment increased % alternation in Y-maze in both wild-type and hPDE4D mice (*n* = 10 male mice); ^#^*p* < 0.05 and *p < 0.05 versus vehicle-treated hPDE4D and wild-type mice, respectively (Dunnett’s multiple comparison test); H-89, a PKA inhibitor, was administered i.c.v. prior to dosing with BPN14770, ^$$^*p* < 0.001 (Student’s *t*-test) versus hPDE4D mice treated with 0.03 mg/kg BPN14770. **b** Rolipram treatment increased % alternation in Y-maze in both wild-type and hPDE4D mice (*n* = 10 male mice); ^#^*p* < 0.05 and *p < 0.05 versus the vehicle -treated hPDE4D and wild-type mice, respectively (Dunnett’s multiple comparison test). **c** BPN14770 treatment increased discrimination index in NOR in both wild-type and hPDE4D mice (24 h inter-trial interval, *n* = pooled analysis of 9–18 males and 5–9 females per group); ^#^*p* < 0.05 or **p < 0.01 versus vehicle-treated group (Dunnett’s multiple comparison test); ^$^*p* < 0.05 (Student’s *t-*test) versus humanized PDE4D mice treated with 0.03 mg/kg BPN14770. **d** Rolipram treatment increased discrimination index in NOR in both wild-type and hPDE4D (24 h inter-trial interval, *n* = pooled analysis of 8–10 males and 6–10 females per group); *p < 0.05 or ^#^*p* < 0.05 versus the vehicle group of the identical genotype (Dunnett’s multiple comparison test). **e** Single acute treatment with BPN14770 reversed the memory impairment induced by scopolamine in NOR (1 h inter-trial interval, *n* = 9–10 mice per group). **f** Repeated treatment with BPN14770 for 14 days reversed the memory impairment induced by scopolamine in NOR (inter-trial interval, *n* = 10 mice per group). Data are presented as mean ± SEM (*n* = 10 males); *p < 0.05 or **p < 0.01 (Student’s *t*-test) versus vehicle-treated mice; ^$^*p* < 0.05 and ^$$^*p* < 0.01 (Dunnett’s multiple comparison) versus scopolamine-treated mice. **g** A high dose of BPN14770 reduced the ketamine/xylazine-induced anesthesia duration in hPDE4D mice (*n* = pooled analysis of 4–5 males and 4–5 females per group); **p < 0.01 (Student’s *t*-test) versus vehicle-treated wild-type group; ^#^*p* < 0.05 or ^##^*p*< 0.01 (Student’s *t*-test) versus vehicle-treated hPDE4D group

To assess working and spatial memory, mice were dosed with BPN14770 by oral gavage 1 h prior to the Y-maze test. Vehicle-treated mice exhibited intact working and spatial memory, as shown by alternation greater than chance levels (50%). BPN14770 improved % alternation in both genotypes (one-way ANOVA; hPDE4D mice: *F*(3, 36) = 5.207, *p* = 0.0043; wild-type mice: *F*(3, 36) = 2.465, *p* = 0.078) with at least 100-fold difference in the MED, i.e., 1 mg/kg in the wild-type mice (*p* < 0.05) as compared to 0.01 mg/kg in the humanized PDE4D mice (*p* < 0.05; Fig. 4a). Additional studies comparing the cognitive response of wild-type and humanized PDE4D mice to BPN14770 focused on long-term memory. To assess cognitive benefit, mice were dosed with BPN14770 by oral gavage 1 h prior to the 5 min training session and then tested for recall 24 h later. Baseline discrimination by mice exposed to vehicle was low for both genotypes and was not significantly different. BPN14770 improved discrimination in both genotypes (one-way ANOVA; a pooled population of males and females; hPDE4D mice: *F*(3, 61) = 3.151, *p* = 0.0312; wild-type mice: *F*(3, 80) = 3.477, *p* = 0.0197) and also with a 100-fold difference in the MED (Fig. 4c). Additional data analyses revealed that female animals responded similarly to the drug as males under the same dose level in the NOR, but the results were more variable (Supplementary Figure S1). In separate experiments, plasma exposure to BPN14770 was determined to be 35 ± 4 ng/ml in C57bl/6 mice 1 h after an oral dose of 0.03 mg/kg; exposure was not affected by genotype (data not shown).

Rolipram was used as a reference compound due to its lack of subtype selectivity. The binding pose of rolipram accommodates either UCR2 phenylalanine or tyrosine, and hence the compound has similar potency against wild-type (IC_50_ = 12 ± 1.5 nM) and humanized mouse PDE4D (IC_50_ = 36 ± 1.5 nM; Table 1). Rolipram improved both % alternation and discrimination of the novel object in both genotypes of mice, with no difference in the dose response comparing wild-type and humanized PDE4D mice (Y-maze for hPDE4D mice: *F*(3, 36) = 3.380, *p* = 0.0286; Y-maze for wild-type mice: *F*(3, 36) = 2.527, *p* = 0.0728; NOR for hPDE4D mice: *F*(3, 67) = 2.193, *p* = 0.0434; NOR for wild-type mice: *F*(3, 63) = 3.117, *p* = 0.0323; Fig. 4b, d). The MED for rolipram was 0.1 mg/kg IP in both genotypes of mice in both tests (all *p* < 0.05). These data provide additional confidence that the greater sensitivity of the humanized PDE4D mice to BPN14770 is due to engagement of the PDE4D target. Importantly, the approximate 100-fold greater potency for BPN14770 in humanized vs. wild-type mice, which is consistent with differences in binding potency, suggests that the PDE4D subtype is the predominant mediator of the effect of this inhibitor on memory. Other PDE4 subtypes may additionally contribute to the regulation of bulk cAMP levels in brain and to LTP in the hippocampus [48, 49], which may account for a smaller shift (approximately 10-fold) in BPN14770 dose response in humanized compared to wild-type mice.

To further explore signaling through the PKA–CREB pathway in the context of PDE4D inhibition, a PKA inhibitor (H-89) was co-administered with BPN14770 [50]. H-89 was dosed by intracerebral ventricular administration through an indwelling cannula (1 µg) followed by oral dosing with BPN14770 (0.03 mg/kg PO). H-89 significantly antagonized the effect of BPN14770 on memory (Fig. 4a, c, *p* < 0.01 and *p* < 0.05, respectively). To evaluate whether intracerebroventricular (i.c.v.) administration of vehicle or drug affected results, two additional groups that received oral vehicle plus i.c.v. vehicle, and oral BPN14770 (0.03 mg/kg) plus i.c.v. vehicle, were also included. However, data from these two groups are not different than data from the groups that received vehicle and BPN14770 (0.03 mg/kg) alone, respectively. Therefore, we chose to present only the latter two for comparison.

The effects of BPN14770 on early memory processes in humanized PDE4D mice were also examined using the scopolamine-induced memory impairment model (Fig. 4e, f). Scopolamine is an antagonist of muscarinic receptors that are positively coupled to adenylyl cyclase [51]. This transiently impairs early stages of memory formation, which can be reversed by compounds that increase cholinergic tone such as the cholinesterase inhibitors used for the treatment of mild-to-moderate Alzheimer’s disease [52]. In the model, scopolamine was used to impair novel object recognition with a 1 h inter-trial interval to assess short-term memory. Untreated mice exhibited intact short-term memory (d2 = 0.3–0.4) while treatment with scopolamine 30 min prior to training abolished short-term memory formation. Acute administration of BPN14770, 30 min prior to scopolamine, dose-dependently reversed the memory deficit (one-way ANOVA, *F*(3, 34) = 2.983, *p* = 0.0448) with an MED = 0.03 mg/kg (*p* < 0.05; Fig. 4e). Thus, the benefit of BPN14770 for short- and long-term memory occurs within a similar range of dose and exposure. Moreover, the memory benefit of BPN14770 in the scopolamine impairment model is maintained with repeated dosing (one-way ANOVA, *F*(3, 36) = 4.556, *p* = 0.0083, Fig. 4f).

Separate experiments verified the pharmacological profile of BPN14770 in outbred ICR (Institute of Cancer Research) mice, with similar memory-enhancing effects observed in the NOR model (Supplementary Figure S2). By contrast, BPN14770 was ineffective in models assessing antidepressant-like (Supplementary Figure S3) or anxiolytic-like (Supplementary Figure S4) effects. This behavioral profile is similar to that of a second, chemically unrelated, allosteric inhibitor of PDE4D and suggests a primary role for PDE4D in modulating the PKA–CREB pathway outflow relevant to memory and cognition.

### Evaluation of a pharmacological correlate of emesis in mice treated with BPN14770

Nausea and emesis are the major side effects that limit the tolerability of PDE4 inhibitors in a therapeutic setting [53]. Rodents lack an emetic reflex as they cannot relax the crural sling, a band of muscle around the neck of the esophagus that allows the vomiting of their stomach contents. Robichaud et al. [53] introduced the duration of ketamine/xylazine-induced anesthesia as a pharmacological surrogate for assessment of emetic potential as commonly seen in other species. PDE4B and PDE4D gene-deleted mice differ by this measure, with PDE4D gene-deleted being more prone to “emesis” by this surrogate [54]. Comparison of wild-type and humanized PDE4D mice demonstrated that BPN14770 dose-dependently reduced the duration of ketamine/xylazine anesthesia (one-way ANOVA; hPDE4D mice: *F*(3, 28) = 5.483, *p* = 0.0043; wild-type mice: *F*(3, 36) = 1.858, *p* = 0.1543) at an MED = 3 mg/kg PO (*p* < 0.01), as shown in Figure 4g. The 1 mg/kg rolipram significantly reduced the anesthesia duration in both genotypes. However, without a full dose–response test, the MED of rolipram for the two genotypes cannot be determined. In separate experiments, plasma exposure at the NOAEL (no observed adverse effect level) of 1 mg/kg PO was 1305 ± 113 ng/ml at 1 h post dose (data not shown). Cognitive benefit and elevation of PKA–CREB pathway biomarkers were shown in the experiments described above to occur at plasma exposures of 10–30 ng/ml. Thus, there is a 40- to 100-fold window in exposure between doses of BPN14770 in the humanized PDE4D mice that benefit neurochemical and cognitive biomarkers of PKA–CREB pathway outflow as compared to the plasma exposure predicted to cause emesis in non-rodent species.

### Expression of hippocampal plasticity-related proteins after repeated exposure to BPN14770

**Fig. 5:**
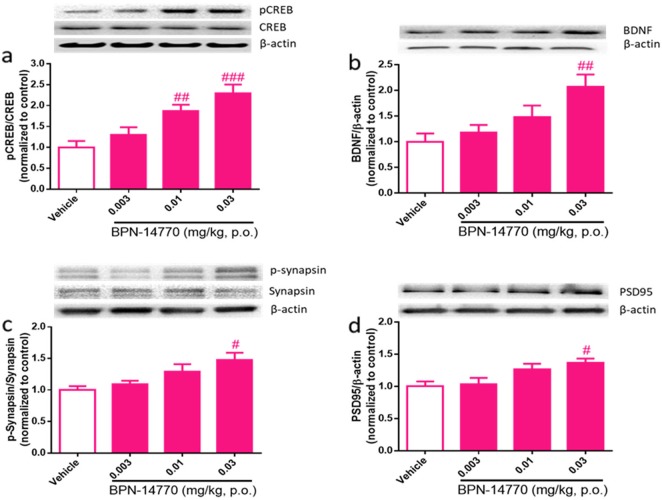
Effects of repeated exposure to BPN14770 on synaptic-related proteins in the hippocampus of humanized PDE4D mice. Humanized PDE4D mice were dosed orally for 14 days and hippocampal samples were collected 1 h after the last dose (*n* = pooled sample of 3–5 males and 3–5 females per group). **a** Phospho-CREB, CREB, and β-actin immunoblot and the calculated pCREB/CREB ratio normalized to vehicle-treated mice. **b** BDNF and β-actin immunoblot with the calculated BDNF/β-actin ratio normalized to vehicle-treated mice. **c** Phospho-synapsin, synapsin, and β-actin immunoblot with the calculated pSynapsin/synapsin ratio normalized to vehicle-treated mice. **d** PSD95 and β-actin immunoblot with the calculated PSD95/β-actin ratio normalized to vehicle-treated mice. Data are presented as mean ± SEM; ^#^*p* < 0.05, ^##^*p* < 0.01, and ^###^*p* < 0.001 (Dunnett’s multiple comparison) versus vehicle-treated group

Neurochemical biomarkers of PKA–CREB activation in humanized PDE4D mice were assessed after acute or repeated exposure to BPN14770. Although single, acute dosing with BPN14770 elevates cAMP and improves short-term and working memory, no change was seen in hippocampal CREB phosphorylation or expression of brain-derived neurotrophic factor (BDNF) at 1 h after dosing (data not shown). With repeated dosing of 14 days of duration, however, dose-dependent elevation of CREB phosphorylation and BDNF expression occurred (one-way ANOVA; pCREB: *F*(2, 23) = 10.29, *p* = 0.0002; BDNF: *F*(2, 24) = 5.415, *p* = 0.0055 Fig. 5a, b); CREB phosphorylation was elevated 2.3-fold (*p* < 0.005) and BDNF was elevated 2.1-fold (*p* < 0.005). The effective doses were consistent with the MED for cognitive improvement in tests of short- and long-term memory.

Repeated dosing with BPN14770 also elevated neurochemical markers of synaptic plasticity in the hippocampus. As a direct target of PKA and an indirect downstream effector of BDNF, synapsin regulates presynaptic vesicle clustering and neurotransmitter release [55]. Repeated dosing with BPN14770 increased phosphorylation of synapsin (one-way ANOVA, *F*(3, 21) = 4.880, *p* = 0.0099) 1.5-fold at a dose of 0.03 mg/kg (Fig. 5c). Synapsin phosphorylation is an indication of enhanced synaptic activity associated with increased synaptic vesicle exocytosis upon neuronal stimulation [56]. Repeated dosing with BPN14770 also increased levels of postsynaptic density 95 (PSD95) 1.4-fold (one-way ANOVA, *F*(3, 21) = 4.892, *p* = 0.0098) at a dose of 0.03 mg/kg (Fig. 5d). PSD95 is located almost exclusively in the postsynaptic density of neurons, and is as an important scaffold protein that helps anchor other synaptic proteins [57]. It plays an important role in the development of synapses and stabilization of synaptic changes during LTP [58]. In a separate study, increased phosphorylation of synapsin was also observed in the prefrontal cortex and cortex of hPDE4D mice after repeated dosing (data not shown). Taken together, these results indicate that inhibition of PDE4D was sufficient to induce both pre- and postsynaptic remodeling and instigate long-lasting changes in synaptic connectivity.

## Discussion

Mice engineered to carry a humanized PDE4D gene provide a unique and powerful genetic model in which to assess the importance of PDE4D as a modulator of PKA–CREB pathway outflow, a fundamental mechanism underlying early and late stages of memory. Uniquely to primates, a key selectivity residue in the PDE4 UCR2 provides a means to design subtype-selective inhibitors of PDE4D [19]. Compounds accessing that binding pose further show selectivity for dimeric versus monomeric forms of PDE4D, and selectivity for the PKA-activated versus the basal state of the PDE4D dimer.

Our studies comparing the responses of wild-type and humanized PDE4D mice to a selective PDE4D allosteric inhibitor demonstrate that PDE4D is a key modulator of PKA–CREB pathway outflow. Selective engagement of the PDE4D target by a single, acute dose of BPN14770 was demonstrated to increase brain cAMP, augment the extended phase of LTP, reverse scopolamine impairment of short-term memory, and improve long-term memory through a PKA-dependent mechanism. Most importantly, BPN14770 was found to be at least 100-fold more potent for its effects in the Y maze and NOR tests in humanized mice compared to wild-type mice. The potency difference was notably less for its neurochemical and electrophysiological effects. This suggests that the memory-enhancing effects observed are likely mediated predominantly by PDE4D, while the other effects are likely mediated by other PDE4 subtypes in addition to PDE4D.

The memory benefit of BPN14770 was maintained with repeated dosing with elevation of hippocampal CREB phosphorylation and BDNF expression. Hippocampal synapsin phosphorylation and PSD95 levels as markers of synaptic remodeling were also elevated after repeated dosing with BPN14770. The effect of BPN14770 on neurochemical and cognitive biomarkers of PKA–CREB pathway outflow occurred across a consistent range of dose (0.01–0.03 mg/kg PO) and plasma exposure (10–30 ng/ml). Although rodents are unable to vomit, a surrogate measure of emesis projects BPN14770 tolerability in non-rodent species of greater than 1300 ng/ml in plasma. Previous work has suggested an association of PDE4D and emetic effects of PDE4 inhibitors. The weak emetic actions of BPN14770 could be due to the predominant expression of short-form PDE4D variants in the area postrema, an emetic trigger region; these variants lack the N-terminal structures required for high affinity binding of the allosteric inhibitor [59].

Given the low exposures needed for benefit and the relatively high exposures projected to lack tolerability, the therapeutic target of BPN14770 is likely the PKA-activated, dimeric form of PDE4D. We speculate that lack of tolerability at high exposures may be due to loss of selectivity for dimeric versus monomeric forms of PDE4D (selectivity window = 16-fold), loss of selectivity for the PKA-activated versus basal state of dimeric forms of PDE4D (selectivity window = 130-fold), or loss of selectivity for PDE4D versus other PDE4 subtypes (selectivity window = 260-fold). BPN14770 is >1000-fold selective against other PDE, off-target G protein-coupled receptors, hERG, transporters, and ion channels (data not shown).

Our studies of PDE4D target engagement by BPN14770 are consistent with previous studies of PDE4 inhibitors with modest selectivity for PDE4D and knockdown of PDE4D messenger RNA (mRNA) expression in mouse brain. In mouse, rolipram is about sevenfold selective for PDE4D versus PDE4B. The compound shows broad benefit across multiple memory paradigms as well as benefit in a mouse model of Alzheimer’s disease (APP_swe_/PS1_M146L_ mice). In the Alzheimer’s disease model, rolipram shows persistent benefit in hippocampal LTP, synaptic spine remodeling, and cognitive endpoints after washout of the drug, consistent with an enduring effect on synaptic stabilization [41]. GEBR-8a and GEBR-32a show 2–3-fold selectivity for PDE4D over other PDE4 subtypes and have also been shown to increase hippocampal cAMP and enhance memory performance in transgenic AD mice with reduced potential for emesis [60, 61]. While our data suggested that reduced emetic-like effects during PDE4D inhibition could be attributed to targeting of specific isoforms of PDE4D, those full inhibitors of PDE4D also have low emetogenic effects, which might be due to their unknown interactions outside the catalytic domain. Knockdown of PDE4D mRNA improves memory performance in mice, and the effects are not enhanced further by rolipram [62]. Amyloid-β42-induced memory deficits are also reversed by knockdown of hippocampal PDE4D mRNA [63].

The use of the humanized PDE4D mouse model provided powerful insight into the role of PDE4D in modulating the PKA–CREB pathway, a fundamental mechanism of early and late stages of memory. BPN14770 has entered human clinical trials for the treatment of memory disorders such as Alzheimer’s disease, as well as other dementias, and in an orphan disorder in Fragile X syndrome, a neurodevelopmental disorder in which cAMP signaling is impaired with consequent intellectual disability and autistic spectrum disorder [64]. PDE4D inhibitors may also be useful for the treatment of psychiatric disorders in which cognition is impaired including schizophrenia, bipolar disorder, and major depression [65].

## Electronic supplementary material

Figure S1

Figure S2

Figure S3

Figure S4

Supplemental Materials and Methods

Supplemental information
