# Supplementary figures and images for: Memory enhancing effects of BPN14770, an allosteric inhibitor of phosphodiesterase-4D, in wild-type and humanized mice
Source: Neuropsychopharmacology. 2018 Aug 14;43(11):2299–309. doi: 10.1038/s41386-018-0178-6 (PMC6135860; doi:10.1038/s41386-018-0178-6)

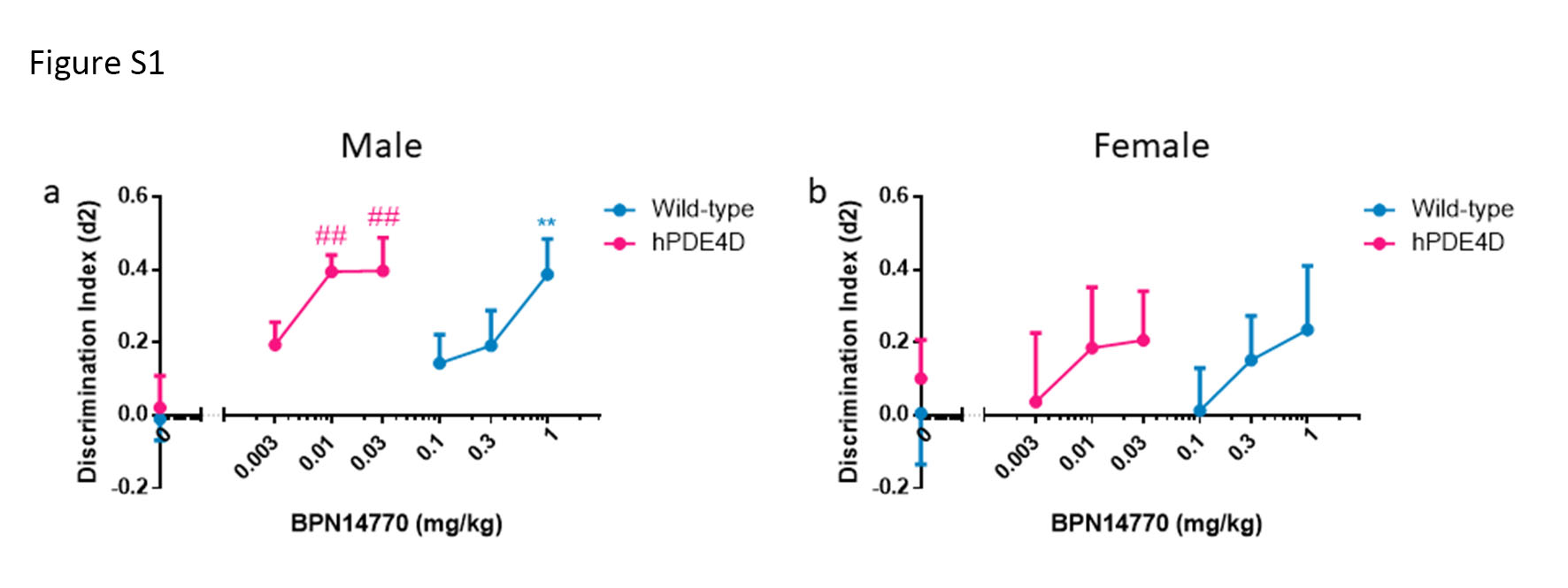

Supplement: Supplementary file 1 — Figure S1 [file 41386_2018_178_MOESM1_ESM.jpg]

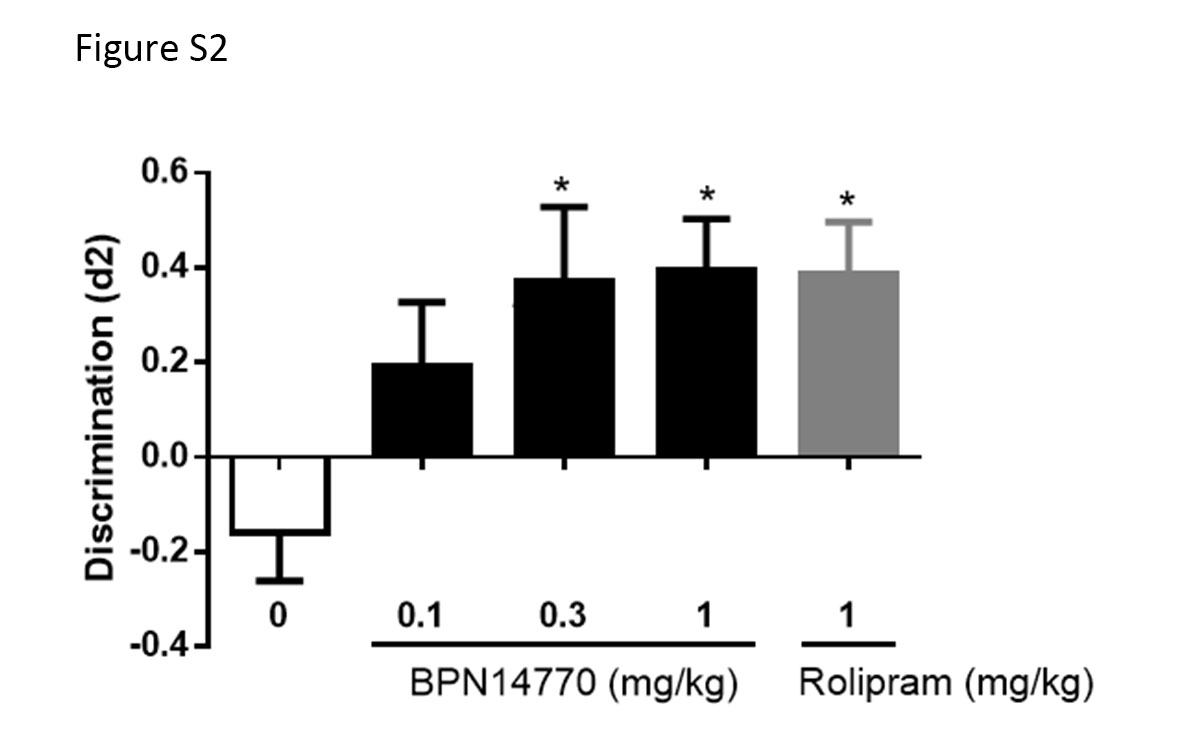

Supplement: Supplementary file 2 — Figure S2 [file 41386_2018_178_MOESM2_ESM.jpg]

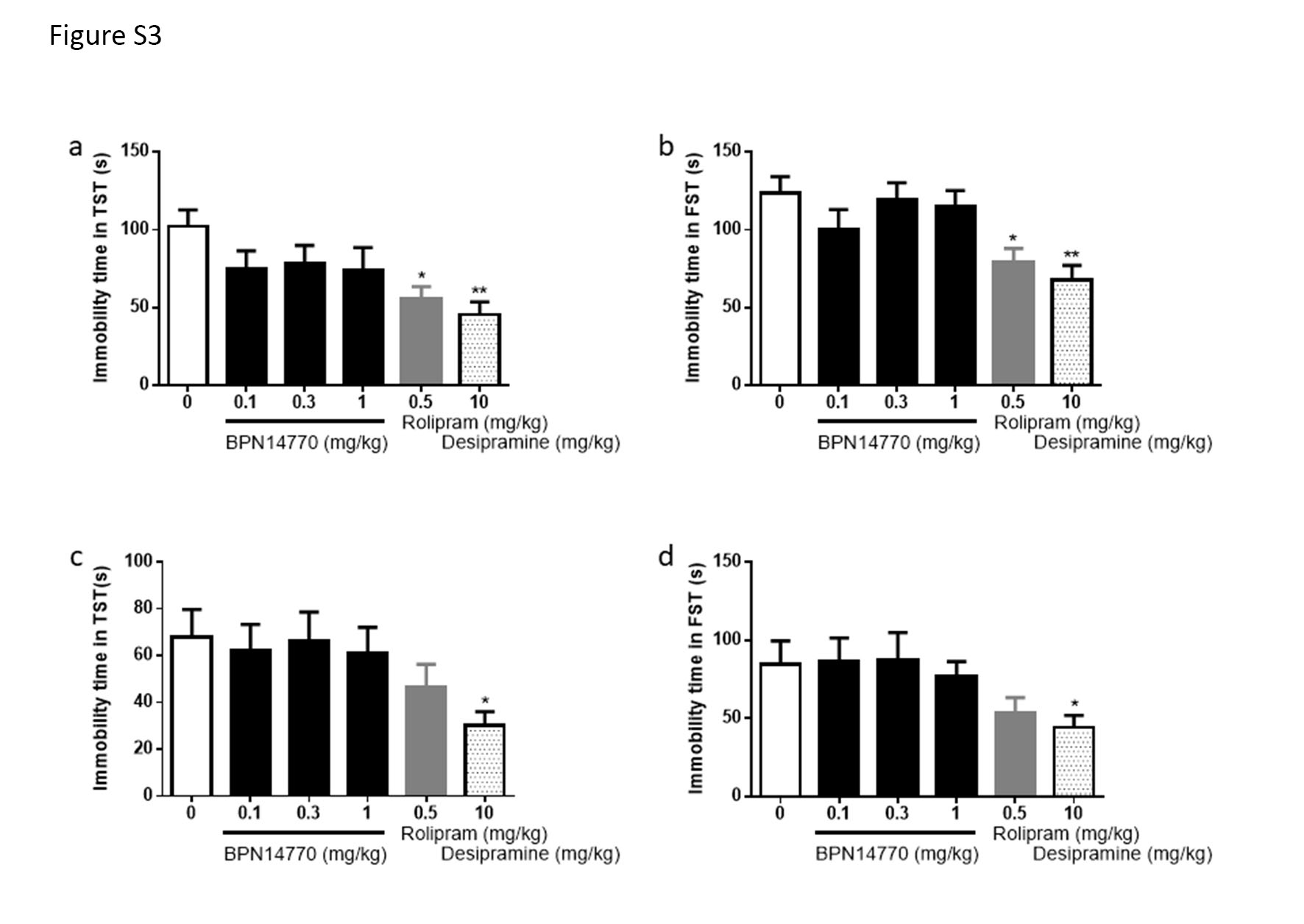

Supplement: Supplementary file 3 — Figure S3 [file 41386_2018_178_MOESM3_ESM.jpg]

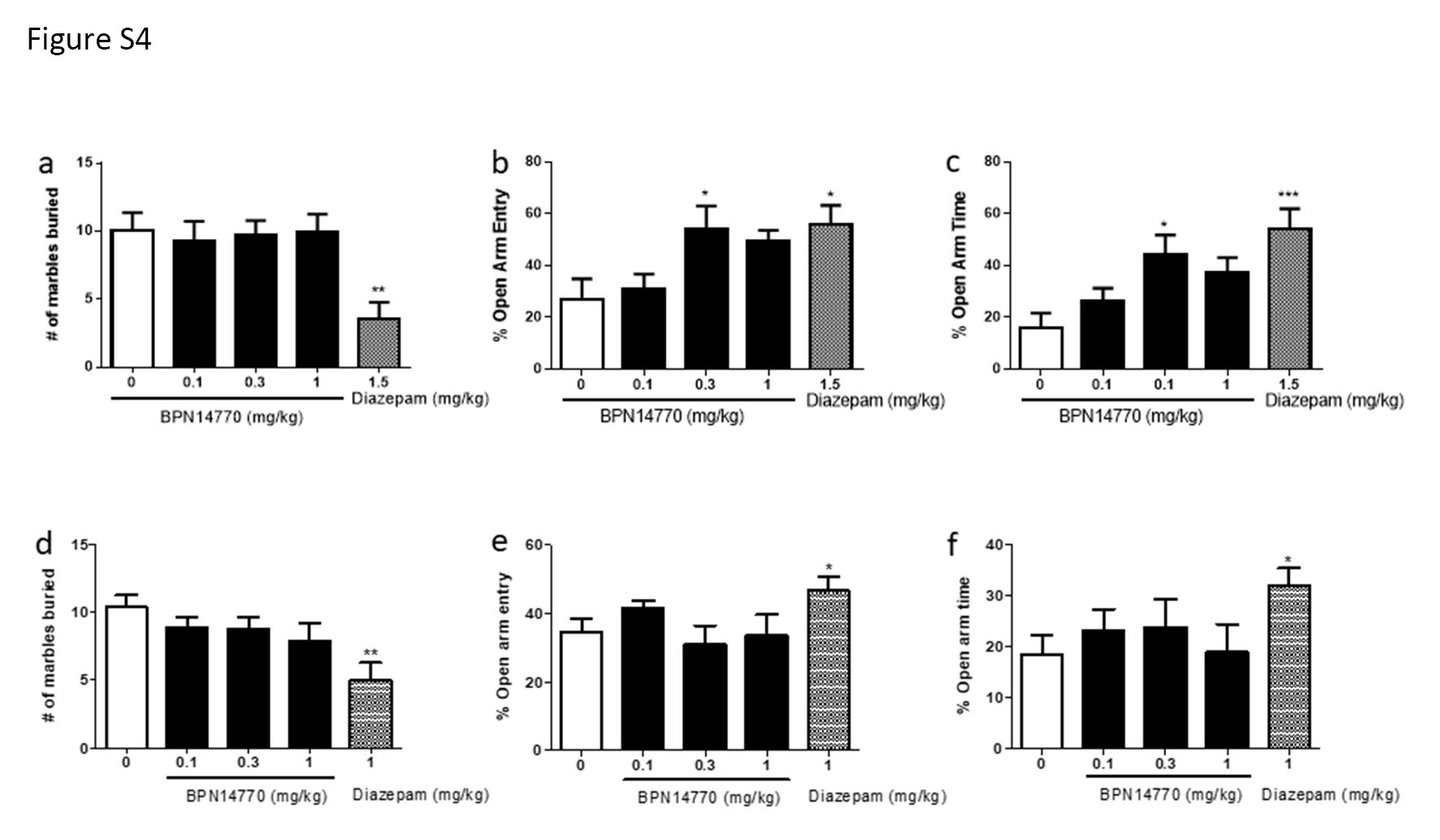

Supplement: Supplementary file 4 — Figure S4 [file 41386_2018_178_MOESM4_ESM.jpg]
