# Supplementary material for: Memory enhancing effects of BPN14770, an allosteric inhibitor of phosphodiesterase-4D, in wild-type and humanized mice
Source: Neuropsychopharmacology. 2018 Aug 14;43(11):2299–309. doi: 10.1038/s41386-018-0178-6 (PMC6135860; doi:10.1038/s41386-018-0178-6)
Supplement: Supplementary file 5 — Supplemental Materials and Methods [file 41386_2018_178_MOESM5_ESM.docx]

**SUPPLEMENTARY MATERIALS AND METHODS**

**Compounds**

BPN14770 was synthesized as described.^58^ Rolipram was purchased from Enzo Life Sciences (Farmingdale, NY). H-89 and scopolamine were purchased from Sigma-Aldrich (St. Louis, MO). [^3^H]-rolipram was purchased from PerkinElmer (Waltham, MA).

**Enzymes**

PDE4D residue numbering is based on the reference PDE4D7 isoform, NCBI Reference Sequence: NP_001159371.1. Methods used to generate synthetic genes for human and mouse PDE4 subtypes and isoforms were as described in Burgin et al.^19^ Synthetic genes were engineered with carboxyl- or amino-terminal hexahistidine tags for Baculovirus-infected insect cell expression and purification. Large scale preparations of infected Sf9 insect cells were grown in ESF921 medium (Expression Systems, Davis, CA). Approximately 2.5 × 10e^6^ Sf9 cells per ml were infected with 5-10% final virus and harvested 40-72 hrs post-infection. Cells were centrifuged to collect cell paste which was frozen drop-wise into liquid nitrogen and stored at -80°C. Frozen cell paste of the PDE4D proteins was resuspended and lysed hypotonically in 20mM tris(hydroxymethyl)aminomethane (Tris) pH 8.0 containing one complete protease inhibitor tablet for 1 hr at 4°C. The lysate was clarified by centrifugation at 42,000 rpm for 45 minutes at 4°C. Sodium chloride (NaCl) was added to the clarified lysate to obtain a final concentration of 0.5M. Clarified lysate was first purified by immobilized metal affinity chromatography with a HiTrap™ Chelating HP column and an AKTA FPLC system. The protein was eluted over a gradient with 20mM Tris pH 8.0, 0.5M NaCl and 0.5M imidazole and the eluted fractions were analyzed via SDS-PAGE prior to pooling. PDE4 proteins were dialyzed into the final buffer containing 20mM Tris at pH 8.0, 0.1M NaCl, 0.1mM MgCl_2_, 0.1mM ZnCl_2_, and 2mM dithiothreitol (DTT) and then flash frozen with liquid nitrogen in aliquots of 0.5 ml at 0.1mg/ml as measured by A_280_. All proteins were stored at -80°C. Purity was assessed by SDS-polyacrylamide gel electrophoresis. The purity of PDE4D7 enzymes was similar and in the range of 60-70% with the major contaminant the truncated catalytic domain lacking UCR1 and UCR2. Contamination by the truncated catalytic domain does not affect measurement of IC_50_ for PDE4 allosteric inhibitors. Vendors were Beryllium Discovery Corp (Bainbridge Island, WA) and Proteos Inc. (Kalamazoo, MI).

**Animals**

C57BL/6 mice homozygous for the humanized PDE4D gene were generated by inGenious Targeting Laboratory (Ronkonkoma, NY). The mouse PDE4D gene was humanized by knocking the tyrosine257 to phenylalanine mutation into exon 7 of the mouse gene in C57BL/6 embryonic stem cells using a targeting vector that allowed for positive/negative selection using a Pgk/Neo cassette. Vector sequences were excised using Flp recombinase, the resultant embryonic stem cells were used for blastocyst injection, and ultimately mice containing the humanized PDE4D gene were bred to homozygosity. As a control, littermate C57BL/6 mice that did not contain the humanized PDE4D gene were bred through brother-sister crosses. Wild-type littermates and humanized PDE4D mice at 8-10 weeks old were used for all behavioral experiments. Mice were kept in a temperature-controlled room under standard laboratory conditions, with a 12 h light/12 h dark cycle, and with free access to food and water. All behavioral tests were performed during 9:30 am–16:30 pm and in accordance with the “NIH Guide for the Care and Use of Laboratory Animals” (revised 2011) and were approved by the Institutional Animal Care and Use Committee of the University at Buffalo.

**PDE4 Biochemical Assays**

Kinetic assay of cAMP hydrolysis by purified PDE4 enzymes is measured by coupling the formation of the PDE4 reaction product 5′-adenosine monophosphate (AMP) to the oxidation of reduced nicotinamide adenine dinucleotide (NADH). This is accomplished with three coupling enzymes (yeast myokinase, pyruvate kinase, and lactate dehydrogenase), and allows fluorescent determination of reaction rates.^19^ Assays were performed in 96-well plates in a total volume of 200 μl/well. Compounds were dissolved in dimethylsulfoxide (DMSO) and added to plates in a volume of 10 μl followed by addition of 165 μl of assay mix. Plates were pre-incubated at 25 °C for 10 min. The enzyme reaction was initiated by the addition of 25 μl of cAMP followed by thorough mixing. Reaction rates were measured by monitoring the decrease in fluorescence using excitation at 355 nm and emission at 460 nm for a period of 10 min in a fluorescence plate reader. Initial rates (slopes) were determined from linear portions of the progress curves. Final concentrations of assay components were as follows: 50 mM Tris, pH 8, 10 mM MgCl_2_, 50 mM KCl, 2% DMSO, 5 mM tris(2-carboxyethyl)phosphine (TCEP), 0.4 mM phosphenolpyruvate (PEP), 0.01 mM NADH, 0.04 mM adenosine triphosphate (ATP), 0.004 mM cAMP, 7.5 units myokinase from yeast, 1.6 units pyruvate kinase, 2 units lactate dehydrogenase, and ~0.5-4 nM PDE4. The concentration of purified PDE4 in the assay was adjusted to yield an initial rate of approximately -0.6 RFU/sec. To measure inhibition of PDE4 enzymes by test compounds, data were percent normalized relative to controls (negative controls: 12 wells per 96 well assay plate with no added cAMP; positive controls: 12 wells with cAMP but no inhibitor) and are presented as percent inhibition. Z’ quality factors were > 0.5 for the assay plates. An Inhibitory Concentration 50% (IC_50_) values were calculated by fitting a sigmoidal dose response curve with I_min_ = 0% inhibition and I_max_ as a fitted parameter, and reported as mean ± S.E.M.

**Protein Structure Determination by X-ray Crystallography**

Co-crystals of PDE4D with BPN5004 were grown by sitting drop vapor diffusion at 289K using 0.4 µl of 5.0 mg/ml protein (in 10mM 4-(2-hydroxyethyl)-1-piperazineethanesulfonic acid (HEPES), pH7.5, 100mM NaCl, 1mM dithiothreitol (DTT), 0.1mM zinc chloride, 0.1mM magnesium chloride) in the presence of 0.5 mM BPN-5004 (DMSO) and combined with 0.4 µl of crystallization buffer containing 8.75% w/v PEG 1,000, 6.25% w/v PEG 3,350, 6.25% v/v 2-methyl-2,4-pentanediol (MPD), 15mM sodium nitrate, 15mM disodium hydrogen phosphate, 15mM ammonium sulfate, 50mM 3-(N-morpholino)propanesulfonic acid (MOPS), 50mM HEPES, pH7.5, 20% v/v ethanol, 50mM phosphate-citrate, pH4.2. Crystals grown were directly flash frozen for data collection. A 1.70Ǻ dataset of a co-crystal of human PDE4D-UCR2 (CID7620) and compound BPN-5004 was collected at the Advanced Photon Source synchrotron, Beamline 21-ID-F (LS-CAT) with a Marmosaic 225 CCD detector. Diffraction data were reduced and scaled with XDS/XSCALE ^59^. The structure of PDE4D-UCR2 bound to BPN5004 was solved by rigid body refinement using pre-existing structures of PDE4D catalytic domain. The structure was refined using iterative cycles of TLS and restrained refinement with REFMAC5 ^60^, part of the CCP4 program suite ^61^, and model-building using the Crystallographic Object-Oriented Toolkit (COOT) ^38^. The structure was peer-reviewed internally and validated using Molprobity^39^ prior to deposition in the Protein Data Bank (PDB: 6BOJ) ^62,63^.

**[^3^H]-Rolipram radioligand binding**

Mice were killed by decapitation, and the brains were immediately dissected on ice and homogenized in binding buffer (50 mM Tris-HCl, 5 mM MgCl_2_, pH 7.5) using a Polytron homogenizer (Brinkman Instruments, Westbury, NY). The membrane fraction of the homogenate was obtained by centrifugation at 15,000 g for 15 min, and resuspension of the pellet in the binding buffer^29^.

For each genotype, mouse brain membrane preparations containing 200 to 300 μg of protein were incubated in duplicate at 30°C in 250 μl of binding buffer containing either i) 0.5-50 nM [^3^H]-rolipram for the saturation binding studies; or ii) various concentrations of BPN14770 and 2 nM [^3^H]-rolipram for the competition studies. Nonspecific binding was defined in the presence of 10 µM unlabeled rolipram. At the end of the incubation, reactions were stopped by adding 5 ml of the ice-cold binding buffer and rapid vacuum filtration through glass fiber filters that had been soaked in 0.3% polyethyleneimine. The filters were then washed twice with 5 ml of the ice-cold binding buffer, and discs of filter paper were collected for measurement of radioactivity by liquid scintillation counting.

**Behavioral tests**

For each of the behavioral tests, BPN14770 was dissolved in a vehicle composed of 5% DMSO, 5% Solutol, and 90% water, and administered orally. Rolipram was dissolved in saline containing 5% DMSO and administered intraperitoneally as a reference compound. For the scopolamine-induced memory impairment models, a single dose of scopolamine (0.1 mg/kg, IP) was administered 30 min before the training session of the NOR. Animals treated with H-89 or vehicle were implanted with 26-gauge guide cannula unilaterally (AP −0.2 mm from Bregma, ML +1 mm from the midline, DV−2mm from skull surface) and rested for a week before any tests. H-89 was prepared in saline and administered to mice 30 min before dosing with BPN14770 using a 33-gauge injection cannula dispensing 5 µl over 5 min. For repeated treatment studies, mice were orally dosed with BPN14770 for 14 days, and the behavioral tests were conducted 1 h after the last dose.

*Y-maze spontaneous alternation test.* The Y-maze apparatus made of matt blue Plexiglas had three identical arms (*L 40 cm, W 5 cm, H 15 cm*) placed at 120° from each other. The test consisted of a single 5 min trial, in which the mouse was allowed to explore all three arms of the Y-maze. The start arm was varied between animals to avoid placement bias. Spontaneous Alternation (%) was defined as consecutive entries in 3 different arms, divided by the number of possible alternations (total arm entries minus 2). Mice with less than 8 arm entries during the 5-min trial were excluded from the analysis.

*Novel object recognition.* Mice were handled for 3-5 min for 5 days in a row. The novel object recognition test was performed in a Plexiglas open field box (*L30 cm, W50 cm, H40 cm*) as described elsewhere^64,65^. Briefly, the task procedure consists of three phases: habituation, training (T1), and testing (T2). In the habituation phase (*day 1*), each animal is allowed to freely explore the apparatus for 10 min; then the animal is returned to its home cage. During the training phase (*day 2*), a single animal is placed in the center of the open field containing two identical objects located on the diagonal of the field, and allowed to explore for 5 min. After a retention interval after T1 of 24 h (long-term recall) or 1 h (scopolamine-impairment model), the animal was subjected to the T2 testing phase for 5 min, during which one familiar object and one novel object were presented. The arena and objects were thoroughly wiped with 70% ethanol after each trial to avoid the presence of any olfactory cues. The animals were considered to be exploring the object when directing their nose to the object at a distance of no more than 2 cm and/or touching or sniffing the object. The duration each animal spent exploring the objects was recorded. Time spent exploring the identical objects in T1 was recorded as *a_1_* and *a_2_*; time spent exploring the familiar and the novel objects in T2 was recorded as ‘*a*’ and ‘*b*’, respectively. The following variables were calculated: *e1 = a_1_ + a_2_, e2 = a + b*, the relative discrimination index *d2 = (b – a)/e2*.

*Ketamine-xylazine test.* A mixture of 8 mg/ml ketamine and 1 mg/ml xylazine was prepared in 0.9% saline on the day of experiment. During the test, mice were dosed with BPN14770 or vehicle (PO), or rolipram (1 mg/kg, IP). 30 min or 15 min (for PO and IP doses, respectively) later, mice were injected (IP) with a mixture of ketamine and xylazine at a dose of 80 mg/kg ketamine and 10 mg/kg xylazine. Once the animals become ataxic, they were placed in the supine position in V-shaped metal troughs. The duration of anesthesia was determined as the time between the loss and return of the righting reflex, and was used as an endpoint to measure the duration of anesthesia. During anesthesia the mice received optical ointment to prevent dryness and irritation in the eyes^65,66^.

**cAMP assay**

Concentrations of cAMP in mouse brain samples were measured using an enzyme-linked immunosorbent assay (ELISA). The mice were treated with various doses of BPN-14770 or vehicle. 1 h after dosing, mice were killed by rapid decapitation, and brains were immediately were dissected on ice, flash frozen in liquid nitrogen, and stored at -80 ̊C. On the day of assay, brain samples (one hemisphere) were ground to a fine powder under liquid nitrogen in a stainless steel mortar, followed by addition of 10 volumes of 0.1 M HCl. Tissue homogenates were then centrifuged at 800ˣg for 15 min. Supernatants were collected for immediate assay or stored frozen for assay later using the cAMP complete ELISA kit (Enzo Life Sciences, Farmingdale, NY) according to the assay protocol ^66^.

**Hippocampal CA1 field recording**

Hippocampi from eight- to ten-week-old wild-type and humanized PDE4D mice, both males and females, were dissected rapidly in ice-cold oxygenated high-sucrose dissection buffer (HSDB). Transverse hippocampal slices at 350 μm thickness were prepared by a compresstome slicer (Precisionary Instruments, Greenville, NC, USA) in the same HSDB. The slices were allowed to recover in the artificial cerebral spinal fluid (aCSF) for at least 1 h at 30°C before recording. In the experiment, the recording chamber was continuously perfused with oxygenated aCSF at a rate of 2 ml/min at 30 °C. A platinum-iridium cluster stimulating electrode (FHC, Bowdoin, ME) was used to stimulate the Schaffer collateral pathway and a glass recording electrode filled with 2 M NaCl (1-2 MΩ) was placed in the stratum radiatum of area CA1 to record field excitatory postsynaptic potential (fEPSP). An input–output curve was used to set the baseline fEPSP at 30–40% of maximal slope, and at least 20 min of baseline recording was obtained to assure the stability of the slice preparation. Long-term potentiation (LTP) was induced by high-frequency stimulation (HFS) consisting of a single train of 100 pulses delivered at 100 Hz. This HFS paradigm was used to induce a short-lasting form of LTP in the control slices, so that any additional potentiation of LTP because of drug infusion could be observed. BPN-14770 was dissolved in DMSO and diluted into aCSF with a final DMSO concentration of 0.01%, and was bath-applied in the flowing aCSF from 10 min before to 30 min after the HSF ^67^.

**Immunoblot Analyses**

Hippocampal tissues were lysed with RIPA buffer containing protease and phosphatase inhibitors and centrifuged at 13,000 rpm for 30 min at 4 °C. Aliquots of supernatant containing 10-30 μg protein were separated using 10% SDS-PAGE as described previously^66^. Proteins from the gels were transferred to polyvinylidene difluoride membranes, blocked with blocking buffer (phosphate buffered saline containing 3% BSA and 0.1% sodium azide), and incubated with primary antibodies overnight at 4°C (phospho-CREB at Ser 133 (# ab32096, diluted 1:1000, abcam, Cambridge, MA), CREB (# sc-186, diluted 1:200, Santa Cruz Biotechnology, Dallas, Texas), β -actin (Catalog # ab8227, diluted 1:2000, abcam, Cambridge, MA)), Synapsin (#2312, diluted 1:1000, Cell Signaling Technology), Phospho-synapsin (#2311, diluted 1:000, Cell Signaling Technology ), and PSD-95 (#2507, diluted 1:1000, Cell Signaling Technology, Danvers, MA). Labeled protein bands were detected using the enhanced chemiluminescence (ECL) method and quantified using Quantity One 1-D Analysis Software.

**Statistical Analyses**

All data are presented as means ± standard errors of mean (SEM) and were analyzed using GraphPad Prism. For multiple comparisons, data were analyzed using one-way or two-way analysis of variance (ANOVA) with Dunnett’s correction for multiple comparisons. Student’s t-test was used for pairwise comparisons between vehicle-treated and positive control drug-treated, or scopolamine-treated groups. Statistical significance was set at *p* < 0.05.
