# Supplementary material for: Memory enhancing effects of BPN14770, an allosteric inhibitor of phosphodiesterase-4D, in wild-type and humanized mice
Source: Neuropsychopharmacology. 2018 Aug 14;43(11):2299–309. doi: 10.1038/s41386-018-0178-6 (PMC6135860; doi:10.1038/s41386-018-0178-6)
Supplement: Supplementary file 6 — Supplemental information [file 41386_2018_178_MOESM6_ESM.docx]

**Table S1:** X-ray diffraction data and refinement statistics.

| **Protein**  **Compound** | PDE4D – UCR2  BPN-5004 |
| --- | --- |
| **Data Collection** |  |
| Space Group | *P2*_1_ |
| Cell dimensions |  |
| *a, b, c* (Å)  α, β, γ (°) | 81.78, 81.89, 116.79  90.000, 110.413, 90.000 |
| Resolution (Å) | 1.70 (1.74-1.70) |
| *R_merge_* (%) | 8.5 (49.9) |
| *I/σ(I)* | 11.85 (3.05) |
| Completeness (%) | 97.3 (95.8) |
| Redundancy | 5.2 (5.1) |
| **Refinement** |  |
| Resolution (Å) | 1.70 |
| No. reflections | 154,546 |
| *R_work_ / R_free_* overall | 16.1 / 18.6 |
| No. atoms  Protein  Ligand  Water | 10755  130  1132 |
| ADP (Å^2^)  Protein  Ligand  Water | 21.17  18.61  31.54 |
| r.m.s. deviations  Bond lengths (Å)  Bond angles (°) | 0.014  1.400 |

**Figure S1 Effects of BPN14770 on memory in the novel-object recognition (NOR) test humanized (hPDE4D) and wild-type PDE4D mice (C57Bl/6), separated by sex.**  Male (a) or female (b) mice were dosed orally with BPN14770 1 hr prior to training; memory was tested 24 hr later. Data are presented as mean ± S.E.M. (n = 9-18 mice per group); ** *p* < 0.01 (Dunnett’s multiple comparison test) versus vehicle-treated wild-type group; ## *p* < 0.05 (Dunnett’s multiple comparison test) versus vehicle-treated hPDE4D group.

**Figure S2 Effects of BPN14770 on memory in the novel object recognition (NOR) test using outbred ICR mice.** Mice were orally dosed with BPN14770 at 1 hr prior to training, or with rolipram immediately after training; memory was tested 24 hr later. Data are presented as mean ± S.E.M. (n = 8 male mice); * *p* < 0.05 (Dunnett’s multiple comparison test) versus vehicle-treated group.

**Figure S3 | Test for potential antidepressant-like effects of BPN14770 in ICR mice.** (a) Tail suspension test performed at 1 hr after acute treatment with BPN14770 (PO), or 30 min after acute treatment with Rolipram (IP) or positive control drug desipramine (IP). (b) Forced swim test performed at 1 hr after acute treatment with BPN14770, or 30 min after acute treatment with Rolipram or positive control drug desipramine. (c) Tail suspension test performed at 1 hr after the last dose of 14-day treatment with BPN14770, 30 min after the last dose of 14-day treatment with Rolipram, or 30 min after acute treatment with the positive control drug desipramine. (d) Forced swim test performed at 1 hr after the last dose of 14-day treatment with BPN14770, 30 min after the last dose of 14-day treatment with Rolipram, or 30 min after acute treatment with the positive control drug desipramine. Data are presented as mean ± S.E.M. (n = 10 male mice); * *p* < 0.05 and ** *p* < 0.01 (Student’s t-test) versus vehicle-treated group.

**Figure S4 |** **Test for potential anxiolytic-like effects of BPN14770 in ICR mice.** (a) Marble-burying test performed at 1 hr after acute treatment with BPN14770 (PO), or 30 min after acute treatment with the positive control drug diazepam (IP). (b) % open arm entry and (c) % open arm time in the elevated-plus maze test performed at 1 hr after acute treatment with BPN14770, or 30 min after acute treatment with the positive control drug diazepam. (d) Marble-burying test performed at 1 hr after the last dose of 14-day treatment with BPN14770, or 30 min after acute treatment with the positive control drug diazepam. (e) % open arm entry and (f) % open arm time in the elevated-plus maze test performed at 1 hr after the last dose of 14-day treatment with BPN14770, or 30 min after acute treatment with the positive control drug diazepam. Data are presented as mean ± S.E.M. (n = 10 male mice); * *p* < 0.05 and *** *p* < 0.001 (Student’s t-test) versus vehicle-treated group.
